# Evaluation of the prognostic values of solute carrier (SLC) family 39 genes for patients with lung adenocarcinoma

**DOI:** 10.18632/aging.202452

**Published:** 2021-02-01

**Authors:** Heng Zhou, Yaoqi Zhu, Huizhong Qi, Liang Liang, Hao Wu, Jingping Yuan, Qingyong Hu

**Affiliations:** 1 Department of Pathology, Renmin Hospital of Wuhan University, Wuhan 430060, Hubei Province, P. R. China; 2 Department of Stomatology, TaikangTongji Hospital, Wuhan 430000, Hubei Province, P. R. China; 3 Jingchu University of Technology, Jingmen 448000, Hubei Province, P. R. China; 4 Department of Phychiatry, Renmin Hospital of Wuhan University, Wuhan 430060, Hubei Province, P. R. China; 5 Department of Oncology, Renmin Hospital of Wuhan University, Wuhan 430060, Hubei Province, P. R. China

**Keywords:** LUAD, NSCLC, SLC39A, SLC39A7, prognosis

## Abstract

Background: Lung cancer is the first fatality rate of cancer-related death worldwide. This study aimed to evaluate the solute carrier family 39 (SLC39A) genes as biological markers associated with the prognosis of lung adenocarcinoma (LUAD).

Methods and materials: MRNA expression of SLC39A genes in non-small cell lung cancer (NSCLC) was analyzed using UCSC database. We investigated the overall survival (OS) of SLC39A genes in patients with NSCLC as the only prognostic indicator using the Kaplan-Meier plotter. CERES score obtained from the Project Achilles was used to perform the survival analysis. Crystal violet-glutaraldehyde solution staining and CCK-8 assay were used to determine colony formation and cell viability, respectively.

Results: For patients with lung squamous cell carcinoma, only high expression of SLC39A3, SLC39A4 and SLC39A7 have significant affections to the prognosis. But for patients with LUAD, 11 out of 14 SLC39A genes were significantly associated with prognostic values. Additional analysis indicated that SLC39A7 played an essential role for cell survival of LUAD. Furthermore, SLC39A7 high expression in LUAD was associated with current smoking.

Conclusions: Our findings indicated that SLC39A groups were significantly associated with prognosis of LUAD. The SLC39A7 gene was significantly linked with survival and growth of LUAD cells.

## INTRODUCTION

Lung cancer is the second morbidity of total diagnosed cancer (13% of the total cases) and the first fatality rate of cancer-related deaths (23% of the total cases). [1] Non-small cell lung cancer (NSCLC) occupies vast majority of primary lung cancers. NSCLC are further classified into lung squamous cell carcinoma (LUSC) and lung adenocarcinoma (LUAD). [2] LUAD accounts for about 50% of all lung cancers. [3, 4] The staging system of LUAD suggested by American Joint Committee on Cancer (AJCC) is by far the most effective predicted indicator of prognosis in patients with LUAD and guides clinical treatment strategy. [3] More than 20% of stage I-II patients ultimately develop to recurrence and metastasis, which leads to a 5-year survival rate of less than 15%. [1] These results suggest that the survival predicted indicators at present are deficient and promote us to find more reliable and efficient predicted indicators for early diagnostic detection.

Zinc (Zn) is a critical microelement for cell growth and survival and is an important cofactor of about 300 enzymes. [5] Zn is involved in gene transcription, and cell growth, development, and differentiation as well as control the metabolism of nucleic acid, protein, carbohydrate and lipid. [6] The diffusing of Zn in cells is rigorously regulated by two members of Zn transport proteins: ZnT family (SLC30A) acted as Zn efflux transporters and ZIP family (SLC39A, Zrt- and Irt-like proteins) acted as Zn influx transporter. [7] By delivering Zn from the intracellular stores to extracellular space, ZnT channels decrease the cytosolic Zn concentration, but ZIP controls Zn transport at the reverse direction. [8] ZIP families included 4 subfamilies: type I subfamily (SLC39A9), type II subfamily (SLC39A1–3), gufA (SLC39A11) and LIV-1 subfamily (SLC39A4–8, 10, and 12–14). [9]

SLC39A genes are involved in development and progress in different kinds of malignances, like breast cancer, lung cancer, prostate cancer, esophageal squamous cell carcinoma, hepatocellular carcinoma and colorectal cancer. [10–15] SLC39A8 expression is sufficient to protect lung epithelia against tumour necrosis factor (TNF)-alpha-induced cytotoxicity. Overexpression of miR-183-96-182 with Zn deficiency in food and SLC39A1 down-regulation directly leads Zn loss in prostate and upregulate the risk for prostate cancer. [16] Ras responsive element binding protein 1(RREB1) positively regulates expression of SLC39A3, which leads Zn accumulation and makes contribution to the development and progression of pancreatic adenocarcinoma. [17] Inhibition of SLC39A5 causes cyclooxygenase 2 downregulation and E-cadherin expression increased and reduces growth esophageal cancer *in vivo* and *in vitro*. [18] In addition, previous reports have indicated that some SLC39A genes are associated with prognosis of patients with cancers and could be considered as diagnostic or prognostic biomarkers in these cancers. SLC39A4 is upregulated in pancreatic cancer and positively associated with inferior prognosis of patients in both surgical and fine needle aspiration guided with endoscopic ultrasound specimens. [14] A recent study has assessed the expression level and prognostic values of all SLC39A genes in gastric carcinoma. [19] However, the relationship between the expression profiles and prognosis of the total SLC39A genes and LUAD is still not clear.

In this study, SLC39A groups were differently expressed in LUAD and corresponding normal tissues and significantly associated with prognostic values of LUAD. The SLC39A7 gene played an essential role in survival and growth of LUAD cells.

## RESULTS

### The expression profiles of SLC39A genes in NSCLC

**Figure 1:**
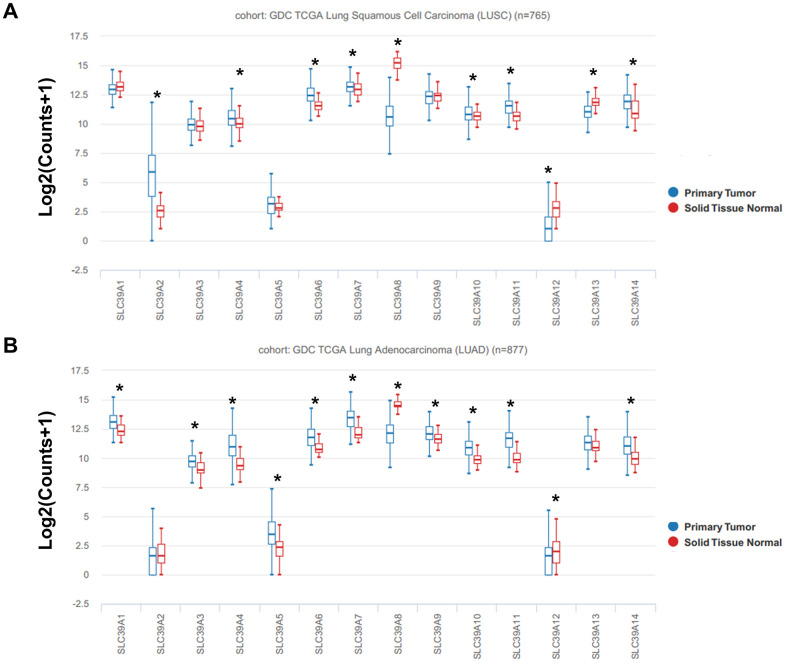
**The box plots showed mRNA expression of total 14 SLC39A families in NSCLC tissues and in normal lung tissues were determined using UCSC database.** Expression of SLC39A1-14 is shown in LUSC (**A**) and LUAD (**B**). * P<0.05; LUSC, lung squamous cell carcinoma; LUAD, lung adenocarcinoma; NSCLC, non-small cell lung cancer; SLC39A, solute carrier family 39.

The mRNA expression profiles of total 14 SLC39A genes in patients with NSCLC from TCGA database was analyzed using UCSC Xena. As shown in Figure 1A, expression of SLC39A2, 4, 6, 7, 10, 11 and 14 was significantly higher in LUSC tissues than which in normal lung tissues, but SLC39A8, 12, and 13 were expressed higher in normal tissues. For patients with LUAD, mRNA levels of SLC39A1, 3, 4, 5, 6, 7, 9, 10, 11 and 14 were significant higher in cancer tissues than in relative normal samples, but SLC39A8 and SLC39A12 were expressed lower in cancer samples. (Figure 1B)

As shown in Supplementary Figure 1, the mRNA expression profiles of SLC39A genes (GSE31210) in patients with LUAD were similar with the results of TCGA-LUAD dataset.

### The prognostic evaluation of SLC39A genes in patients with LUSC

**Figure 2:**
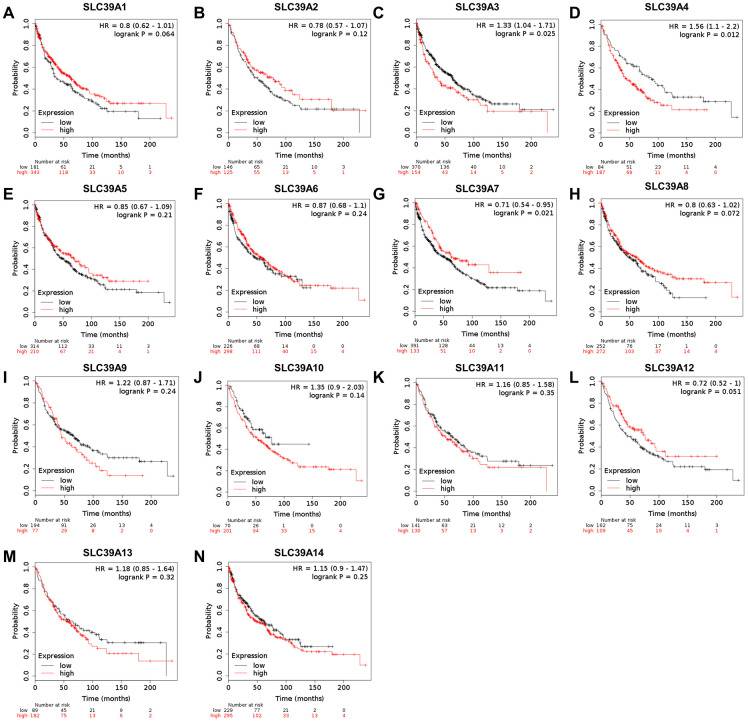
**The prognostic values (OS) of SLC39A members in patients with lung squamous cell carcinoma using Kaplan-Meier plotter.** SLC39A1 – SLC39A14 (**A**–**N**). OS, overall survival; SLC39A, solute carrier family 39.

The prognostic values of SLC39A genes and LUSC were determined by using the Kaplan-Meier plotter with TCGA-LUSC data. As shown in Figure 2, high level of SLC39A3 and SLC39A4 were significantly associated with inferior OS in patients with LUSC. High expressed SLC39A7 was related to better OS in patients with LUSC. Expression of other SLC39A genes, including SLC39A1, 2, 5, 6, 8, 9, 10, 11, 12, 13 and 14 were not associated prognostic values of LUSC.

### The prognostic evaluation of SLC39A genes in patients with LUAD

**Figure 3:**
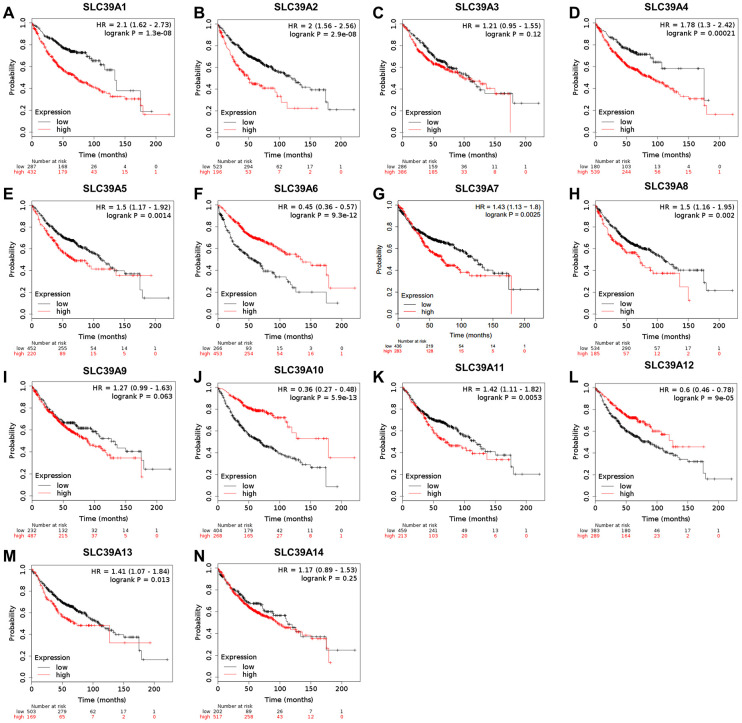
**The prognostic values (OS) of SLC39A genes in patients with lung adenocarcinoma by using Kaplan-Meier plotter.** SLC39A1 – SLC39A14 (**A**–**N**). OS, overall survival; SLC39A, solute carrier family 39.

The same prognostic analysis of SLC39A genes and LUAD were performed. As shown in Figure 3, high expression of SLC39A1, 2, 4, 5, 7, 8, 11 and 13 was significantly associated with inferior prognostic values in patients with LUAD. High expressed SLC39A6, 10 and 12 were significantly linked with preferable OS. Other SLC39A genes including SLC39A3, 9 and 14 showed no significant influence on prognosis of patients with LUAD. By analyzing the dataset of GSE31210, twelve out of fourteen survival results were consistent with TCGA-LUAD except for SLC39A5 and SLC39A10 (P>0.05, Supplementary Figure 2).

### Subgroup analysis of the prognostic evaluation of SLC39A genes in patients with LUAD

For more significant associations were found between prognostic values with expression levels of SLC39A genes in LUAD than LUSC, further explorations of OS with various clinicopathological features in LUAD based on clinical stages, genders and smoking statues were performed. As shown in Table 1, overexpressed SLC39A1, 4, 5, 7, and 11 was significantly associated with more undesirable prognostic value in patients with Stage I LUAD. In contrast, high expression of SLC39A2, 6, 8, 9, 10 and 12 indicated better prognosis. For patients with Stage II LUAD, high expression of SLC39A1, 2, 4 and 5 suggested inferior prognosis, but high expression of SLC39A6, 8, 9, 10, 12 and 14 were associated with better prognosis. In Stage III LUAD patients, only high expressed SLC39A9 suggested poorer prognosis, but high expressed SLC39A2, 4, 12 and 14 were associated with preferable prognosis.

**Table 1:** Overall survival between SLC39A genes expression and stages of lung adenocarcinoma.

**Genes**	**Stage I**			**Stage II**			**Stage III**		
	**Cases**	**HR**	**P**	**Cases**	**HR**	**P**	**Cases**	**HR**	**P**
SLC39A1	370	7.28(3.38-15.7)	3E-09*	136	2.01(1.22-3.31)	0.0051*	24	1.88(0.62-5.65)	0.26
SLC39A2	370	0.61(0.41-0.91)	0.013*	136	3.7(2.22-6.16)	9.1E-08*	24	0.35(0.12-0.98)	0.037*
SLC39A3	346	0.84(0.56-1.26)	0.4	118	1.25(0.71-2.19)	0.44	21	0.52(0.15-1.78)	0.29
SLC39A4	370	2.82(1.76-4.53)	6.5E-06*	136	1.82(1.1-3)	0.017*	24	0.21(0.07-0.63)	0.0026*
SLC39A5	346	1.49(1-2.22)	0.05*	118	2.25(1.32-3.83)	0.0022*	21	2.7(0.76-9.56)	0.11
SLC39A6	370	0.44(0.3-0.65)	1.7E-05*	136	0.34(0.21-0.56)	6.5E-06*	24	0.68(0.25-1.84)	0.44
SLC39A7	370	2.16(1.45-3.22)	0.0001*	136	1.4(0.85-2.31)	0.18	24	0.67(0.24-1.86)	0.44
SLC39A8	370	0.37(0.25-0.55)	2.7E-07*	136	0.33(0.2-0.57)	2.7E-05*	24	2.32(0.52-10.39)	0.26
SLC39A9	346	0.44(0.29-0.66)	4.3E-05*	118	0.44(0.24-0.79)	0.0044*	21	4.6(1.01-20.87)	0.03*
SLC39A10	346	0.22(0.13-0.36)	2.7E-11*	118	0.47(0.28-0.8)	0.0043*	21	5.17(0.66-40.48)	0.081
SLC39A11	346	1.96(1.28-3)	0.0017*	118	1.61(0.95-2.71)	0.072	21	2.19(0.47-10.15)	0.3
SLC39A12	346	0.38(0.26-0.57)	1.1E-06*	118	0.55(0.32-0.96)	0.032*	21	0.22(0.06-0.79)	0.011*
SLC39A13	346	0.69(0.46-1.03)	0.067	118	1.58(0.93-2.71)	0.091	21	2.77(0.56-13.63)	0.2
SLC39A14	370	1.4(0.93-2.11)	0.1	136	0.48(0.29-0.78)	0.0028*	24	2.84(1.02-7.94)	0.038*

*The p value means statistically significant.

Abbreviations: HR, hazard ratio; SLC, solute carrier.

Regarding the genders, SLC39A1, 2, 4, and 11 high expression suggested worse prognostic values, but SLC39A6, 8, 9, 10, 12 and 13 high expression indicated better prognostic information in female group (Table 2). For male patients with LUAD, SLC39A1, 2, 4, and 11 high expression was significantly linked with worse prognosis, but SLC39A6, 7, 8, 9, 10 and 12 high expression indicated more favorable prognosis.

**Table 2:** Overall survival between SLC39A genes expression and genders of lung adenocarcinoma.

**Genes**	**Female**			**Male**		
	**Cases**	**HR**	**P**	**Cases**	**HR**	**P**
SLC39A1	317	2.88(1.69-4.91)	4.6E-05*	344	2.1(1.44-3.06)	8.6E-05*
SLC39A2	317	1.98(1.34-2.92)	0.0005*	344	1.62(1.13-2.33)	0.0085*
SLC39A3	286	1.24(0.82-1.87)	0.31	328	0.76(0.54-1.08)	0.12
SLC39A4	317	3.38(1.81-6.32)	5E-05*	344	1.48(1.05-2.07)	0.023*
SLC39A5	286	1.33(0.89-2)	0.16	328	1.36(0.96-1.91)	0.079
SLC39A6	317	0.34(0.23-0.5)	8.6E-09*	344	0.57(0.41-0.79)	0.00062*
SLC39A7	317	1.43(0.97-2.09)	0.068	344	0.69(0.48-0.98)	0.038*
SLC39A8	317	0.43(0.29-0.63)	1.3E-05*	344	0.38(0.23-0.6)	2.5E-05*
SLC39A9	286	0.32(0.21-0.48)	5.4E-09*	328	0.48(0.32-0.72)	0.00032*
SLC39A10	286	0.28(0.17-0.46)	6E-08*	328	0.43(0.29-0.65)	3.5E-05*
SLC39A11	286	1.63(1.08-2.45)	0.018*	328	1.45(1.03-2.04)	0.033*
SLC39A12	286	0.37(0.25-0.56)	5.5E-07*	328	0.54(0.37-0.79)	0.0013*
SLC39A13	286	0.65(0.43-0.97)	0.035*	328	0.74(0.52-1.06)	0.095
SLC39A14	317	0.72(0.47-1.08)	0.11	344	1.22(0.88-1.69)	0.23

*The p value means statistically significant.

Abbreviations: HR, hazard ratio; SLC, solute carrier.

According to the smoking status, two groups were set as never smoking and smoking (included smoking history). As shown in Table 3, for never smoking patients, high mRNA expression of SLC39A1, 2, 4, 7, 11 and 13 indicated as inferior OS. While SLC39A8, 9 and 10 mRNA high expression was related with better prognostic value. Regarding to smoking patients, high level of SLC39A1, 2, 4, 5, 7, 11 and 13 suggested more undesirable prognosis, but increased expression of SLC39A6, 8, 9, 10 and 12 indicated better prognosis.

**Table 3:** Overall survival between SLC39A genes expression and smoking statues of lung adenocarcinoma.

**Genes**	**Never smoking**			**Smoking (Including smoking history)**		
	**Cases**	**HR**	**P**	**Cases**	**HR**	**P**
SLC39A1	143	3.92(1.67-9.17)	0.00068*	246	2.07(1.3-3.3)	0.0018*
SLC39A2	143	2.34(1.04-5.28)	0.034*	246	1.72(1.06-2.77)	0.025*
SLC39A3	140	0.41(0.14-1.22)	0.099	231	1.4(0.83-2.36)	0.21
SLC39A4	143	3.01(1.34-6.73)	0.0049*	246	2.54(1.3-4.96)	0.0046*
SLC39A5	140	0.48(0.21-1.08)	0.069	231	1.76(1.09-2.86)	0.02*
SLC39A6	143	0.46(0.2-1.05)	0.059	246	0.43(0.27-0.69)	0.00033*
SLC39A7	143	2.31(1.03-5.21)	0.037*	246	2.05(1.28-2.39)	0.0024*
SLC39A8	143	0.19(0.03-0.63)	0.0024*	246	0.35(0.22-0.55)	3.6E-06*
SLC39A9	140	0.37(0.16-0.83)	0.013*	231	0.4(0.24-0.66)	0.00024*
SLC39A10	140	0.31(0.12-0.85)	0.016*	231	0.34(0.2-0.58)	2.5E-05*
SLC39A11	140	2.73(1.2-6.2)	0.012*	231	1.85(1.13-3.02)	0.013*
SLC39A12	140	0.63(0.27-1.45)	0.27	231	0.44(0.26-0.75)	0.0017*
SLC39A13	140	2.33(1.03-5.29)	0.038*	231	1.9(1.15-3.15)	0.011*
SLC39A14	143	0.62(0.27-1.39)	0.24	246	1.37(0.85-2.23)	0.2

*The p value means statistically significant.

Abbreviations: HR, hazard ratio; SLC, solute carrier.

### SLC39A7 plays an essential role for LUAD cell survival

**Figure 4:**
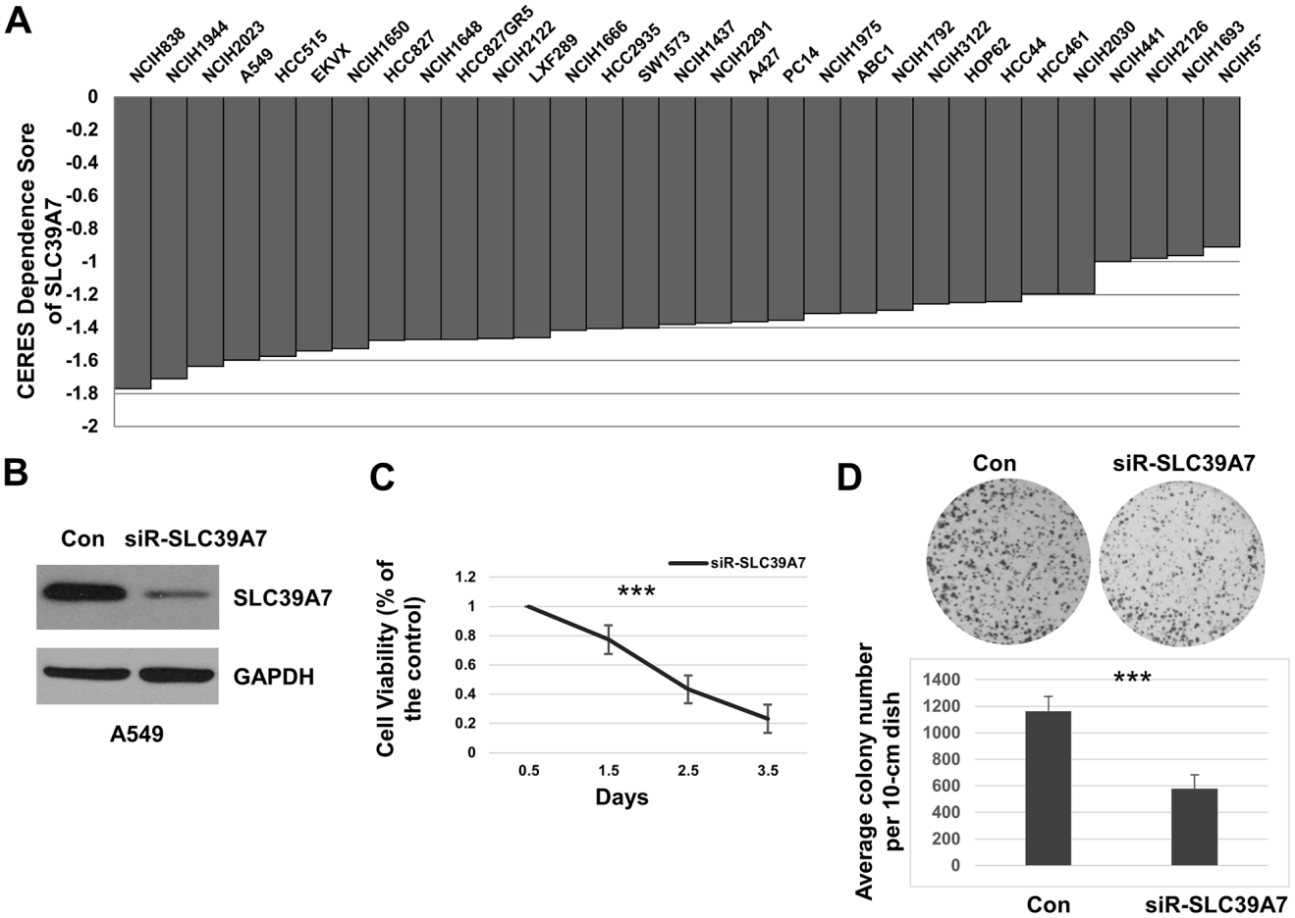
**SLC39A7 played a vital role in cell survival of lung adenocarcinoma.** (**A**) CERES values of SLC39A7 in various lung adenocarcinoma cell lines, the score less than -1 means the gene is important for cell survival. (**B**) After 48 h transfected with control siRNA or siRNA-SLC39A7, A549 cells were harvested. The lysates were undergone for detecting SLC39A7 by Western blotting. (**C**) After seeding, A549 cells were transfected with control siRNA or siRNA-SLC39A7. The CCK-8 assay was performed at 0.5, 1.5, 2.5 and 3.5 days after transfection. The cell viability of siRNA-SLC39A7 treatment was normalized to control group. (**D**) A549 cells were transfected with control siRNA or siRNA-SLC39A7. Then the quantitative cells were placed and cultured for 14 day, and stained with crystal violet-glutaraldehyde solution. All experiments performed in triplicate, and the results are calculated using mean ± standard deviation. One-way ANOVA or Student t test was carried out for evaluating the statistical significance between groups. *** P<0.001. SLC39A, solute carrier family 39.

The survival data of LUAD cell lines was downloaded from Project Achilles. As shown in Figure 4A, the CERES scores suggested total 27 out of 31 LUAD cell lines were less than -1 by silencing SLC39A7 gene expression with the CRISPR-Cas9 system (NCIH838, NCIH1944, NCIH2023, A549, HCC515, EKVX, NCIH1650, HCC827, NCIH1648, HCC827GR5, NCIH2122, LXF289, NCIH1666, HCC2935, SW1573, NCIH1437, NCIH2291, A427, PC14, NCIH1975, ABC1, NCIH1792, NCIH3122, HOP62, HCC44, HCC461, NCIH2030), which demonstrated that SLC39A7 gene is essential for cell survival in LUAD. Conversely, as shown in Supplementary Table 1, other SLC39A genes were not associated with the cell survival of LUAD (CERES scores > -1). To further investigate the relationship between SLC39A7 and cell growth of LUAD, a special knockdown assay targeting SLC39A7 was performed. Western bolt showed SLC39A7 was downregulated with siR-SLC39A7 transfected in A549 cells (Figure 4B). As shown in Figure 4C, the cell viability of A549 cell was significantly inhibited with siR-SLC39A7 treatment over the time (P<0.001). Furthermore, we investigate the role of siR-SLC39A7 in cell colony formation. When knocking down SLC39A7 expression, the size and number of cell colony was significantly inhibited (Figure 4D). Above all, these results indicated an important role played by SLC39A7 for LUAD cell survival.

### High expression of SLC39A7 is associated with smoking

**Figure 5:**
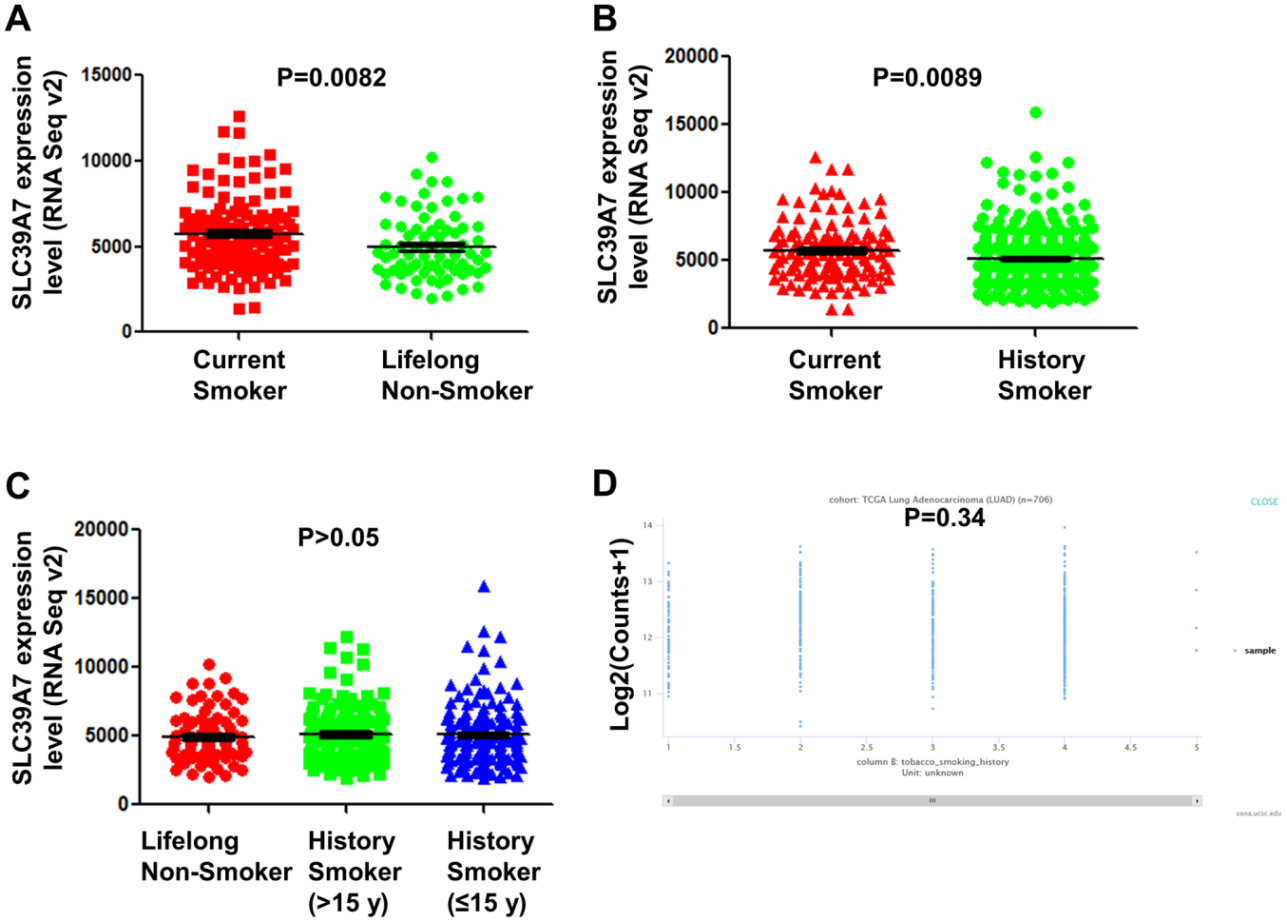
**SLC39A7 expression in lung adenocarcinoma was associated with smoking statues.** TCGA-LUAD dataset was downloaded and analyzed. (**A**) Expression of SLC39A7 in current smokers or lifelong non-smokers. (**B**) Expression of SLC39A7 in current smokers or history smokers. (**C**) Expression of SLC39A7 between lifelong non-smokers, history smokers (>15 y) and history smokers (≤15 y). (**D**) SLC39A7 expression based on smoking duration time (y) analyzed by using UCSC databases. SLC39A, solute carrier family 39.

Smoking is a critical inducing factor for lung cancer. To determine SLC39A7 expression was whether associated with smoking, the TCGA-LUAD data was analyzed. As shown in Figure 5A, 5B, SLC39A7 was found highly expressed in current smoker patients than those never smoking or only having smoking history. However, no significant association was found between lifelong non-smoker, history smoker (>15 y) and history smoker (≤15 y). (Figure 5C) In addition, SLC39A7 level was not associated with the current smoking years. (Figure 5D) These results suggest that expression of SLC39A7 could be provisionally increased by smoking.

### GO enrichment analysis for SLC39A7

**Figure 6:**
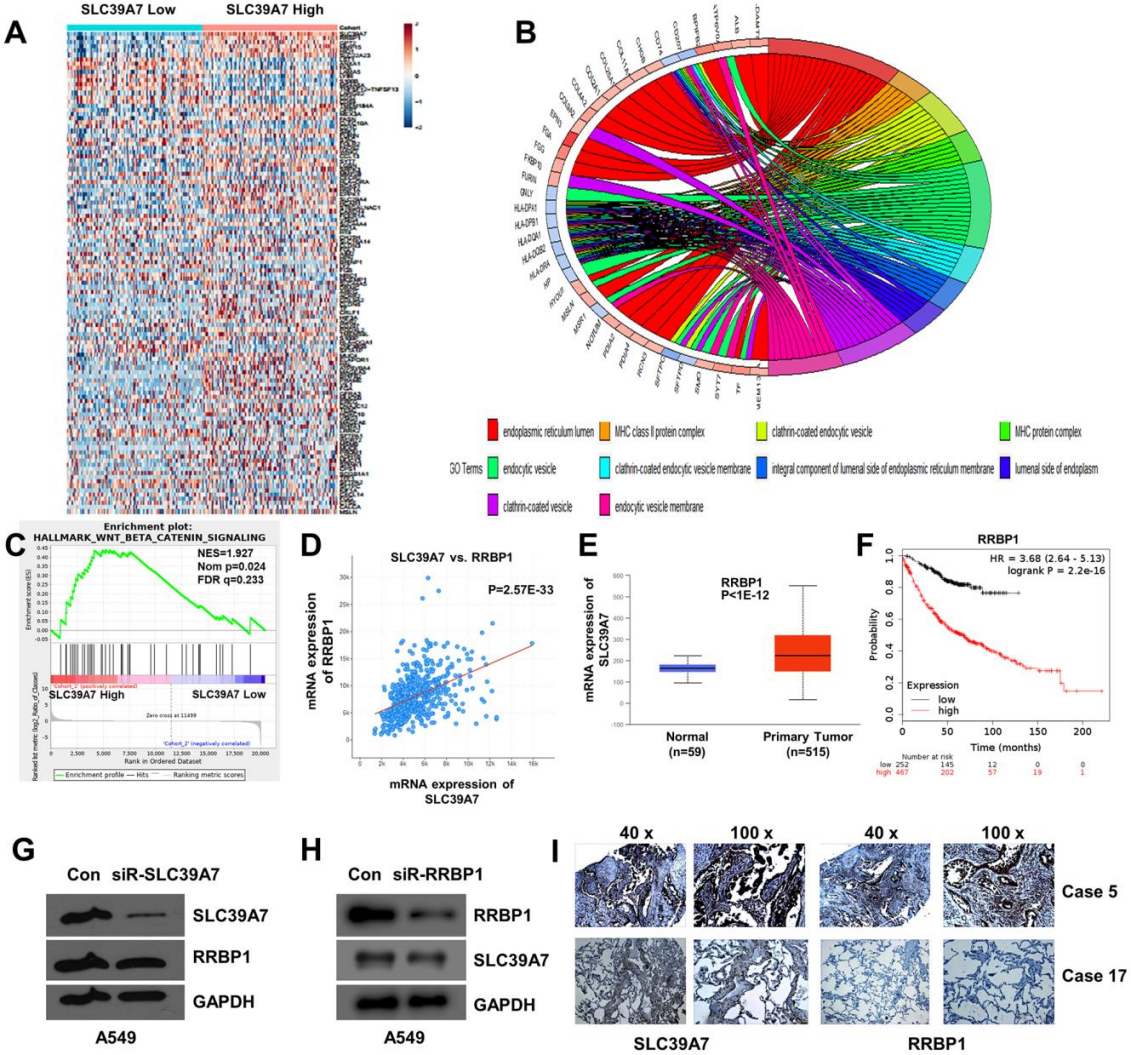
**GO enriched analysis, GSEA analysis and co-expression analysis of SLC39A7 in lung adenocarcinoma.** (**A**) Heat-map showed the DEGs of SLC39A7. (**B**) GO enriched analysis (CC) for SLC39A7. (**C**) GSEA of Wnt/β-catenin signaling pathway. (**D**) Co-expression of SLC39A7 and RRBP1 by using cBioportal database. (**E**) RRBP1 expression of mRNA in LUAD tissues and relative normal tissues in Ualcan database. (**F**) Kaplan-Meier plotter was used to evaluate prognostic value (OS) of RRBP1 in lung adenocarcinoma. A549 cells were transfected with a control siRNA or siRNA-SLC39A7 (**G**)/ RRBP1 (**H**). Western blotting was performed to detect the expression of SLC39A7 and RRBP1. (**I**) Immunohistochemistry assays were performed to detect expression and distribution of SLC39A7 and RRBP1 proteins in lung adenocarcinoma tissues. DEG, differentially expressed genes; GSEA, Gene Set Enrichment Analysis; RRBP1, ribosome binding protein 1; SLC39A, solute carrier family 39.

To explore cell functional change by SLC39A7 expression in LUAD cells, a GO Enrichment Analysis was performed. As shown in Figure 6A, total 301 DEGs were identified according to the expression level of SLC39A7 and the heat-map was given. As shown in Figure 6B, GO enrichment analysis based on cellular component (CC) was given. The most valuable top 10 were endoplasmic reticulum lumen, integral component of lumenal side of endoplasmic reticulum membrane, MHC class II protein complex, MHC protein complex, clathrin-coated endocytic vesicle membrane, clathrin-coated endocytic vesicle, lumenal side of endoplasmic reticulum membrane, clathrin-coated vesicle, endocytic vesicle, and endocytic vesicle membrane.(Table 4) For biological process (BP) analysis, high expression of SLC39A7 mainly enhanced multicellular organismal homeostasis, positive regulation of cell-cell adhesion, and maintenance of gastrointestinal epithelium. (Table 4)

**Table 4:** GO Enriched analysis of DEG based on SLC39A7 expression.

	**Description**	**Gene ratio**	**p.adjust**	**Q-value**
Cellular Component	endoplasmic reticulum lumen	20/272	4.60E-06	3.79E-06
	MHC class II protein complex	6/272	9.79E-06	8.06E-06
	clathrin-coated endocytic vesicle	9/272	2.71E-05	2.24E-05
	MHC protein complex	6/272	7.96E-05	6.56E-05
	endocytic vesicle	17/272	7.96E-05	6.56E-05
	clathrin-coated endocytic vesicle membrane	7/272	0.0001	0.0001
	integral component of lumenal side of endoplasmic reticulum membrane	6/272	0.0001	0.0001
	lumenal side of endoplasmic reticulum membrane	6/272	0.0001	0.0001
	clathrin-coated vesicle	12/272	0.0004	0.0003
	endocytic vesicle membrane	11/272	0.0008	0.0006
	ER to Golgi transport vesicle membrane	7/272	0.0009	0.0007
	clathrin-coated vesicle membrane	8/272	0.0034	0.0027
	basal plasma membrane	5/272	0.0033	0.0027
	coated vesicle	13/272	0.0047	0.0038
	collagen trimer	7/272	0.0061	0.0050
	COPII-coated ER to Golgi transport vesicle	7/272	0.0065	0.0054
	endosomal part	18/272	0.0066	0.0055
	apical plasma membrane	13/272	0.008140053	0.006704555
Biological Process	positive regulation of cell-cell adhesion	16/260	0.004028374	0.003570151
	multicellular organismal homeostasis	18/260	0.004028374	0.003570151
	maintenance of gastrointestinal epithelium	5/260	0.005140237	0.004555541

Ps: p-adjust value < 0.05was considered as significantly enriched.

Abbreviations: DEG, differentially expressed gene; SLC, solute carrier.

### The GSEA analysis of SLC39A7 in LUAD

The Hallmark effect gene sets was evaluated by GSEA analysis. As shown in Figure 6C, the Wnt beta-catenin was significantly associated with SLC39A7 high expression (NES=1.93, NOM p=0.023, FDR q=0.23). Other affected signal pathways included fatty acid metabolism (NES=6.37, NOM p<0.001, FDR q=0.01), estrogen response late (NES=6.38, NOM p=0.0014, FDR q=0.05) and coagulation (NES=4.73, NOM p=0.0014, FDR q=0.027).

### SLC39A7 and RRBP1 were co-expressed in LUAD patients

To further study the potential mechanism of SLC39A7 in LUAD, a co-expressed data was analyzed by using cBioportal database. Totally, 9623 genes were identified with the co-expression profile of SLC39A7 in patients with LUAD, and RRBP1 was a correlated gene. (Figure 6D, p=2.565E-33) Further analysis using UALCAN database indicated that RRBP1 was also high expressed in LUAD tissues than in corresponding normal lung tissues. (P<1E-12, Figure 6E) In addition, over-expressed RRBP1 was associated with poorer prognosis in patients with LUAD. (P=2.2E-16, Figure 6F) To determine the expression relationship between SLC39A7 and RRBP1, siR-SLC39A7 or siR-RRBP1 were transfected into A549 cells for 48 h. As shown in Figure 6G, 6H, knocking down of SLC39A7 cannot inhibit the expression of RRBP1. On the contrary, knocking down of RRBP1 significantly reduce the expression of SLC39A7. To further certificate the above results, we investigate the expression and distribution of SLC39A7 and RRBP1 in LUAD patients. Totally 58 LUAD cases were included and investigated from Renmin Hospital of Hubei Province. As shown in Figure 6I, both SLC39A7 and RRBP1 were mainly expressed in cytoplasm. According to the expression strength of SLC39A7, all patients (58 cases) with LUAD were divided into SLC39A7-High (32 cases) and SLC39A7-Low groups (26 cases). In SLC39A7-High group, 25 samples showed RRBP1-positive. In SLC39A7-Low group, 4 samples presented RRBP1-positive. Protein expression of SLC39A7 was positively correlated with RRBP1 protein (P<0.001). These results suggested that SLC39A7 could be associated with the RRBP1 signaling pathways in LUAD.

### The protein expression of SLC39A genes in LUAD

**Figure 7:**
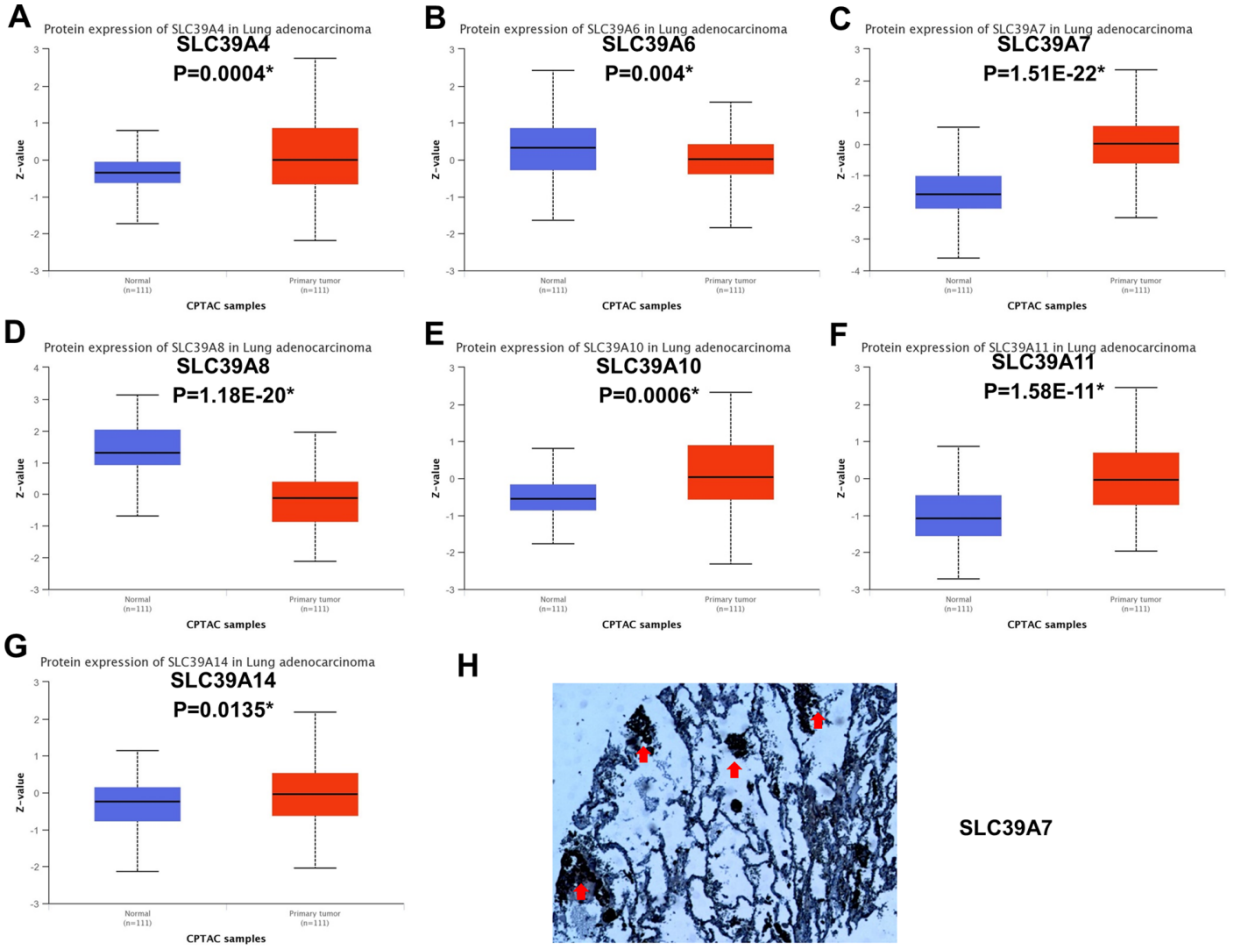
**Protein expression of SLC39A genes in patients with lung adenocarcinoma.** (**A**) SLC39A4. (**B**) SLC39A6. (**C**) SLC39A7. (**D**) SLC39A8. (**E**) SLC39A10. (**F**) SLC39A11. (**G**) SLC39A14. (**H**) Immunohistochemical staining of SLC39A7 proteins in lung adenocarcinoma tissue specimens. * P<0.05; SLC39A, solute carrier family 39.

The mRNA level could not be completed reflect associated protein expression. Therefore, we investigated the protein profiles in LUAD patients from CPTAC by using UALCAN database. SLC39A4, 7, 10, 11 and 14 in cancer tissues were significantly highly expressed compared with in normal tissues.(P<0.05, Figure 7A, 7C, 7E–7G) But, expression of SLC39A6 and SLC39A8 were higher in normal samples than in cancer samples. (P<0.05, Figure 7B, 7D) Other SLC39A genes were not investigated in this database. Except for SLC39A6, other results were consistent with which in mRNA levels. Representative immunohistochemical staining specimen of SLC39A7 in LUAD and normal lung tissue was presented in Figure 7H. The expression of SLC39A7 was significantly higher in tumor tissues than which in normal lung tissue.

## DISCUSSION

Influx and efflux of cellular Zn was temporally and spatially controlled by the ZnT and ZIP families with tissue specific way. [20] Previous studies have reported that many diseases are associated with defect of Zn transporters, such as Alzheimer’s disease, diabetes and malignancies. [21–26] In general, the role Zn played in human is a tumor inhibition agent. Kolenko and his colleagues have reported that the decline of Zn in tumor tissues and serum of patients with prostate carcinoma, as well as decreased expression of SLC39A1, 2, and 3 has been detected. [27] In patients with breast cancer, Zn concentration in serum is lower than which in healthy people, but the association in tissue is inverse. [28] Also, some sporadic reports focusing on the relationship between SLC39A genes and lung cancer indicated that SLC39A4 and SLC39A8 may be an attractive drug target for lung cancer. [10, 29] Hence, we conducted this study to comprehensively evaluate the prognostic associations and expression profiles of SLC39A families in lung cancer.

In this study, we have firstly evaluated the expression levels of SLC39A families and their prognostic correlations in NSCLC using comprehensive bioinformatics methods. Our results indicated that SLC39A2, 4, 6, 7, 8, 10, 11, 12, 13 and 14 were significantly differently expressed in LUSC tissues. For patients with LUAD, mRNA levels of SLC39A1, 3, 4, 5, 6, 7, 8, 9, 10, 11, 12 and 14 have significant difference in cancer tissues than in relative normal samples. For patients with LUSC, only high expression of SLC39A3, 4 and 7 have significant affections to prognosis. But for patients with LUAD, 11 out of 14 SLC39A genes were significantly associated with prognostic values. Additional analysis demonstrated that SLC39A7 gene is critical for LUAD cell survival. High expression of SLC39A7 in LUAD was associated with current smoking state.

High expressed SLC39A4 is found in several malignances and associated with poorer prognosis, including lung cancer. [10, 30] Our findings suggested that the expression of SLC39A4 was high in both LUSC and LUAD, and positively associated with relative worse prognostic values. Although most SLC39A genes were found differently expressed in LUSC samples, the fact only SLC39A3, 4 and 7 have significant affection to prognosis stopped us to conduct a further exploration. For patients with LUAD, SLC39A1, 5, 7 and 11 were significantly highly expressed in cancer tissues than in normal lung tissues, and high expression of associated genes was associated with poorer prognosis. SLC39A2 and SLC39A13 were found not differentially expressed between cancer tissues and normal tissues, but highly expressed of which also indicated poorer prognosis of LUAD patients. SLC39A12 was expressed lower in cancer sample than in normal tissue, and positively associated with a better prognosis of patients in the future. SLC39A6 was highly expressed in cancer tissues, but high expressed SLC39A6 was correlated with a favorable OS of LUAD patients. Different from our findings, Wan et al. have found that PTCH1-3'UTR could attract miR-101-3p approach and increased expression of SLC39A6, and high expressed SLC39A6 was associated with progression of NSCLC. [31] Through subgroup analysis, our results suggested that high level of SLC39A6 predicated good prognosis in different groups, which support the comprehensive results. SLC39A8 expression is involved in the obtaining of inhibition of Cadmium (Cd) in lung cells, indicating an adaptive survival mechanism that resists Cd-induced cytotoxicity. In addition, upregulation of SLC39A8 is sufficient to protect lung epithelia against tumour necrosis factor (TNF)-alpha-induced cytotoxicity. [29, 32] The above results suggested that SLC39A8 in critical for lung cancer cell to resist exogenous stimulation. And our findings suggested that high expression of SLC39A8 suggested an inferior prognosis. The higher expression SLC39A9 was detected in LUAD tissue, but high expression of SLC39A9 is weakly associated inferior outcome of patients. Additional subgroup analysis suggested that for clinical stage I and stage II LUAD patients, high expression of SLC39A9 indicated a preferable OS, but suggested an inferior prognosis for stage III patients, which suggested that SLC39A9 plays various roles in different stages of the disease. Similar situations were found in SLC39A10 and SLC39A14. These results suggested that these SLC39A genes may be available molecular biomarkers for predicating the prognosis of LUAD. SLC39A3 is the only one that be found not associated with different expression and prognosis in LUAD. To confirm the above results, the original data of GSE31210 was analyzed. The expression data of SLC39A genes were consistent with results of TCGA-LUAD. Twelve out of 14 survival analyses were consistent with TCGA-LUAD except for SLC39A5 and SLC39A10. We considered that 3 major points leads the difference. First and the most important, intrinsic heterogeneity of sample originated from race, gender, age.... were hardly avoided. Second, testing platform and method also leaded some difference to the last results. Third, TCGA-LUAD dataset has 877 samples, and GSE31210 only has 225 samples. Larger sample size may reduce the difference. Above all, we considered that the results of TCGA-LUAD were more reliable.

SLC39A7 is critical for cell growth, invasion and migration of several malignances, including breast, cervical, gastric, colorectal and prostate cancers. [15, 33–35] In the present study, we firstly found that SLC39A7 was the only gene which critical for cell survival in all SLC39A genes. Downregulated SLC39A7 significantly suppressed cell growth and cell colony which suggested that SLC39A7 was important for LUAD cell survival. For biological function, high expression of SLC39A7 mainly enhanced multicellular organismal homeostasis, positive regulation of cell-cell adhesion, and maintenance of gastrointestinal epithelium, which may contribute to progress of cancer. By suppressing expression of SLC39A7 and activity of Wnt/β-catenin, miR-15a-3p inhibits cell proliferation and invasion in prostate cancer. [33] Our results suggested that high expression of SLC39A7 associates with activating of Wnt/β-catenin signaling pathway in LUAD tissue which may accounts for inhibition of cell growth. RRBP1 is highly expressed in lung cancer tissues. Knocking down of RRBP1 promotes to stress of endoplasmic reticulum and significantly reduced cell viability and tumorigenicity. [36] In the present study, we found that RRBP1 was accounted for expression of SLC39A7, and high expressed RRBP1 suggested poor prognosis of LUAD. Additionally, through GO enriched analysis, the fact that most DEGs based on differential expression of SLC39A7 were located in endoplasmic reticulum lumen was consistent with this point. The above results suggested that SLC39A7 may be a potential treatment target for LUAD. Importantly, SLC39A7 was higher expressed in current smokers than non-smokers, which suggests SLC39A7 could be transiently increased by smoking. Interestingly, high expression of SLC39A7 indicated poor prognosis in both smokers and non-smokers.

In conclusion, SLC39A families are promising prognostic biomarkers of LUAD except for SLC39A3. The SLC39A7 is essential for survival and growth of LUAD cells. Nevertheless, the evidence on the association between SLC39A families and LUAD is not enough which calls for more attractions to clarify their correlations in the future.

## MATERIALS AND METHODS

*UCSC Xena* UCSC Xena (http://xenabrowser.net/) is an easy-to-use and comprehensive web tool which allows users to perform a customized analysis the data from the Cancer Genome Atlas (TCGA). We evaluated different mRNA expression of SLC39A genes in primary LUSC (TCGA-LUSC, n = 765) and LUAD (TCGA-LUAD, n = 706) in UCSC Xena with a one way ANOVA analysis.

### Kaplan-Meier plotter

Kaplan-Meier plotter (http://kmplot.com/analysis/index.php?p=background) provides prognostic evaluation of more than 54000 genes in total twenty-one cancer types from TCGA databases. [37] The association between overall survival (OS) of patients with NSCLC and mRNA expression profiles of SLC39A genes was analyzed using the Kaplan-Meier plotter. Then, a stratified analysis was performed based on relatively clinicopathological features to determine the association between the OS of patients with LUAD and SLC39A genes with this tool. A logrank P value and hazard ratio (HR) with 95% confidence intervals were calculated in this analysis. To perform the prognostic evaluation, all cases were divided into two groups automatically with selecting best cutoff.

### Cancer virtual cohort discovery analysis platform (CVCDAP) analyzed tool

Key features of CVCDAP (https://omics.bjcancer.org/cvcdap/home.do) is a comprehensive online analyzed tool which contains twenty-nine inbuilt tools which provided analysis of mRNA and protein levels and clinicopathological data in TCGA datasets. The differentially expressed genes (DEGs) and visualized heatmap based on expression levels of SLC39A7 were performed in this web (Log2 fold change=1, FDR=0.05).

### cBioportal for cancer genomics

*The relative* clinical and mRNA expression data of TCGA-LUAD were downloaded for cBioportal for Cancer Genomics (http://www.cbioportal.org/). Additionally, the co-expression data were also analyzed by using cBioportal for Cancer Genomics.

### GEO datasets analysis.

The expression data as well as clinical information of GSE31210 (contain 226 primary LUADs and 20 normal lung tissues) were obtained from Gene Expression Omnibus (GEO) datasets (http://www.ncbi.nlm.nih.gov/gds/) and used to evaluate the relative mRNA expression profiles of SLC39A genes in LUAD patients and normal lung tissues. Sangerbox (http://sangerbox.com/AllTools?tool_id=9698927), a comprehensive tool, was utilized to perform single factor survival analysis (Cox regression).

### Gene set enrichment analysis (GSEA) of SLC39A7

To determine the molecular function of SLC39A7 in LUAD, a GSEA analysis (http://www.broad.mit.edu/gsea) with the Hallmark effector gene sets was performed based on the mRNA level of SLC39A7 in the TCGA-LUAD dataset. [38]

### Gene ontology (GO) enrichment analysis. GO

Enrichment Analysis were performed by using R for Windows version 3.5.2. The library packages included “DOSE”, “clusterProfiler”, “stringr”, and “GOplot”.

### Scores of project Achilles

By using CRISPR-Cas9 technology, Project Achilles systematically explores and identifies numbers of genes which are essential for survival of cancer cell lines, and provides CERES dependence scores. [39] To explore the importance of SLC39A genes for LUAD cell survival, we obtained CERES dependence scores from Depmap portal (https://depmap.org/portal).

### Cell lines and reagents

A549 cell line was kindly provided by Prof. Xiaoping Sun of Wuhan University. The A549 cell line was cultured at humidified atmosphere of 5% CO_2_ and 37° C and grown in DMEM media (HyClone; USA) with 10% FBS (Gibco; USA) and 1% antibiotic (penicillin/ streptomycin, Sigma; USA).

### Cell transfection

The special small interfering RNA (siRNA, SLC39A7, 5’-ACAAGAAAGGCAACAAUUCCAGAAUUGUUGCCUUUCUUGUCG-3’; ribosome binding protein 1(RRBP1), 5’-UGUUUUCUCCUUCUUUUUCUUGAAAAAGAAGGAGAAAACAGU-3’ GenePharma, Co., Ltd.) or a non-specific control was transfected into A549 cells using X-treme GENE HP DNA Transfection Reagent (Roche) according to the manufacturer’s instructions.

### Cell viability assay

A CCK-8 assay (Dojindo Molecular Technologies; Japan) was performed to determine the survival rate of A549 cells treated with or without siRNA-SLC39A7. The A549 cells were added to 96-well plates and cultured in 5% CO2 at 37° C. After seeding, the cells were transfected with siRNA. After 0.5, 1.5, 2.5, 3.5 days transfection, 10 μl CCK-8 solution was added and cultured with these cells at 37° C for 1 h. The OD value was measured at 450 nm by using an absorbance microplate reader (ELx800, BioTek Instruments; USA).

### Colony-formation assay

A549 cells were transfected with control siRNA or siRNA-SLC39A7. Then cells were digested and seeded in 35 mm dishes. After culturing in 5% CO2 at 37° C for 14 days, cells were stained with 0.6% crystal violet-glutaraldehyde solution for 1 min. Lastly, the number of cell colony was counted and photographed.

### Western blot analysis

After 48 h transfected with siRNA, A549 cells were undergone for a Western blot assay. [40, 41] Cells were collected and homogenized in modified RIPA lysis buffer (Beyotime, China) with 0.5% protease inhibitor cocktail (Roche Diagnostics) and stayed on ice for 10 min. After centrifuging at 12,000 rpm, the supernatants were obtained and sonicated for 15 sec. The supernatants were quantified by BCA assay (BioRad Laboratories, USA). Then, the extracts were boiled with 5X loading buffer [Chunfeng Lv, China]. Twenty 20 μg total protein were electrophoresed through 10% SDS-PAGE gels and, then transferred onto the PVDF membrane. After blocking nonspecific binding sites with 5% milk in PBS for 1 h, membranes were incubated with primary antibodies of GAPDH (cat no. 5174; 1:2,000; Cell Signaling Technology, Inc; USA); RRBP1 (cat no. ab224354, 1:1000, Abcam, UK) or SLC39A7 (cat no. ab254566; 1:1000; Abcam, UK) overnight at 4° C. Then, the membranes were cultured with secondary antibodies conjugated with horseradish peroxidase at room temperature for 1 h. Last, the membranes were treated with ECL reagent for developing films at darkness.

### Tissue sample and immunohistochemistry

Totally, 58 cases of LUAD were included in this study. The involved tumor patients were come from the Renmin Hospital of Wuhan University and definitely diagnosed in department of pathology in 2017. Immunohistochemistry was performed to detect the expression and distribution of SLC39A7 or RRBP1 in LUAD. Briefly, the paraffin-embedded sections were deparaffinized, restored and quenched. After blocking with goat serum for 1 h, sections were incubated with primary antibodies against SLC39A7 (cat no. ab254566, 1:1000, Abcam, UK) and RRBP1 (cat no. ab224354, 1:1000, Abcam, UK) overnight at 4° C. Then, the sections were washed with PBS and incubated with secondary antibody (MaxVision™ Kits, MXB, China) with horseradish peroxidase-conjugated polymer for 15 min. Subsequently, tissue section was stained with a DAB for 1 min. Finally, sections were counterstained with Harris hematoxylin for 20 s.

The interpretation of immunohistochemical staining results for SLC39A7 and RRBP1 were performed by two independent and unwitting researchers (HZ and JY) in our group. According to the staining intensity of SLC39A7 in tumor cells, all cases were classified into 4 catalogues as following: scores 0 = lacking, 1 = mild, 2 = moderate and 3 = strong. For analyzing, all patients were divided into two groups according to the scores of SLC39A7: SLC39A7-L (score value = 0, 1 and 2) and SLC39A7-H (score value = 3). The expression levels of RRBP1 were directly divided into two groups: positive group (expressed) and negative group (absent).

### Statistical analysis

All results were calculated with three repeated experiments and presented as mean ± standard deviation (SD). Student t test or one way ANOVA (Newman-Keuls post analysis) was selected to evaluate statistical significance in two groups or more than two groups, respectively. The statistical significance between SLC39A7 and RRBP1 expression profiles in LUAD samples was determined using Chi-square test. The threshold of statistical significance was limited as P-value <0.05. All statistical analysis was performed with SPSS software.

## Supplementary Material

Supplementary Figures

Supplementary Table 1
