# Supplementary material for: Evaluation of the prognostic values of solute carrier (SLC) family 39 genes for patients with lung adenocarcinoma
Source: Aging (Albany NY). 2021 Feb 1;13(4):5312–31. doi: 10.18632/aging.202452 (PMC7950255; doi:10.18632/aging.202452)
Supplement: Supplementary Figures [file aging-13-202452-s001.pdf]

SUPPLEMENTARY FIGURES

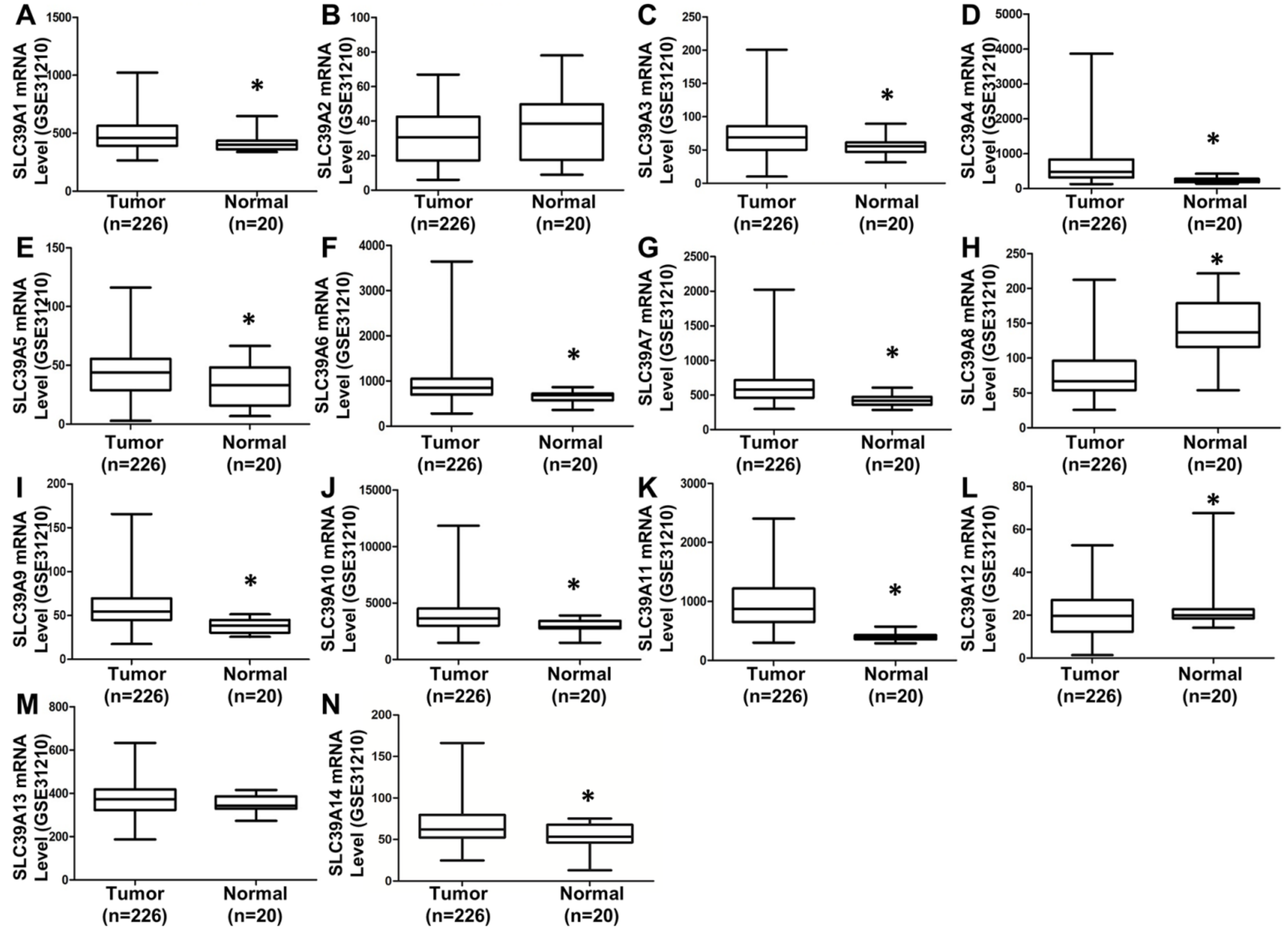

Supplementary Figure 1. The box plots showed mRNA expression of total 14 SLC39A families in lung adenocarcinoma and normal lung tissues by analyzing GSE31210 dataset. (**A**–**N**). SLC39A1 – SLC39A14. * P<0.05; SLC39A, solute carrier family 39.

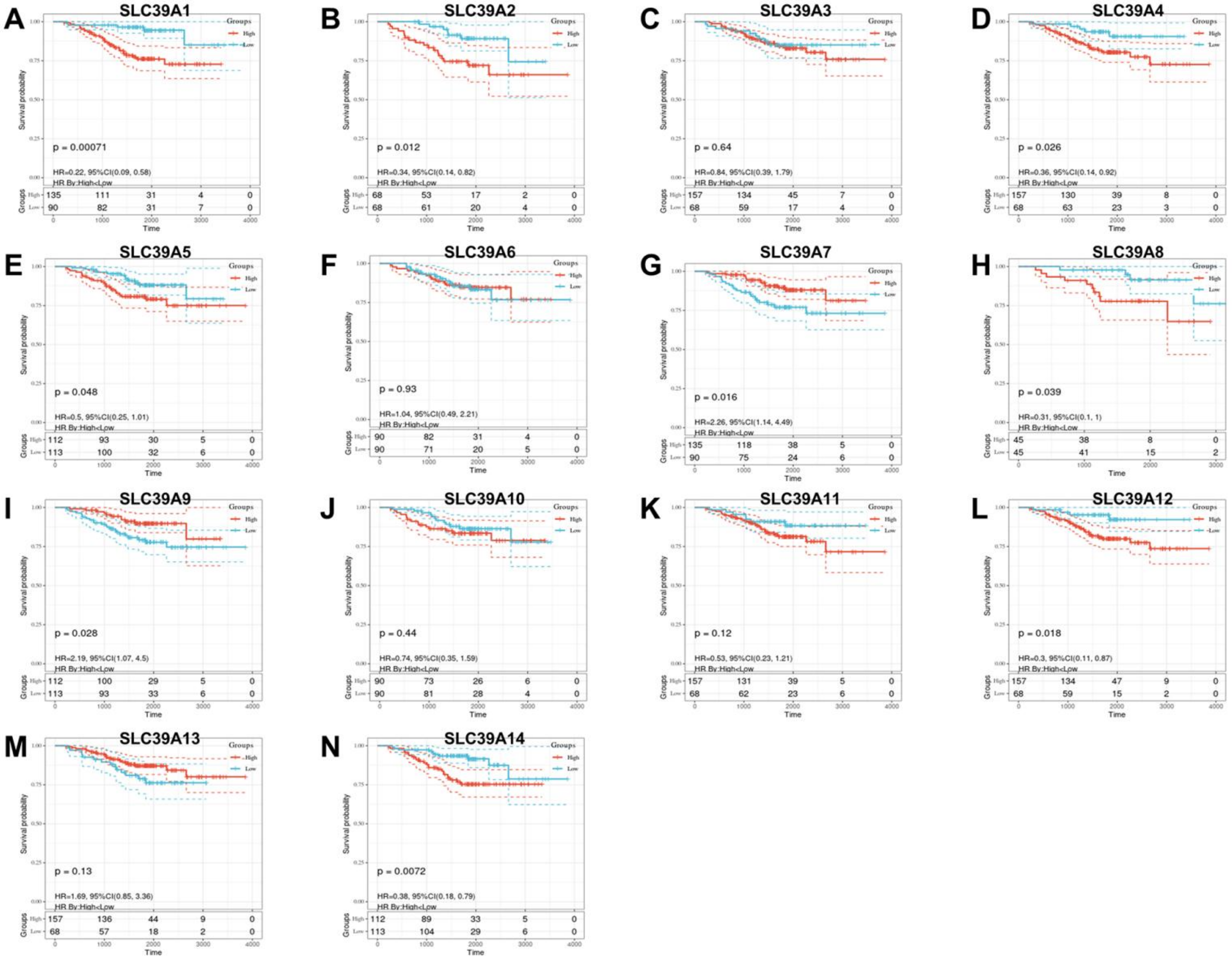

Supplemental Figure 2. Overall Survival of SLC39A families in patients with lung adenocarcinoma by analyzing GSE31210 dataset. (**A**–**N**). SLC39A1 – SLC39A14. SLC39A, solute carrier family 39.
