# Supplementary material for: Evaluation of the prognostic values of solute carrier (SLC) family 39 genes for patients with lung adenocarcinoma
Source: Aging (Albany NY). 2021 Feb 1;13(4):5312–31. doi: 10.18632/aging.202452 (PMC7950255; doi:10.18632/aging.202452)
Supplement: Supplementary Table 1 [file aging-13-202452-s002.docx]

**Supplement Table 1. CERES dependence score of SLC39A genes in lung adenocarcinoma cell lines.**

| Cell Line  Gene Name | SLC39A1 (27173) | SLC39A2 (29986) | SLC39A3 (29985) | SLC39A4 (55630) | SLC39A5 (283375) | SLC39A6 (25800) | SLC39A7 (7922) | SLC39A8 (64116) | SLC39A9 (55334) | SLC39A10 (57181) | SLC39A11 (201266) | SLC39A12 (221074) | SLC39A13 (91252) | SLC39A14 (23516) |
| --- | --- | --- | --- | --- | --- | --- | --- | --- | --- | --- | --- | --- | --- | --- |
| NCIH838 | 0.038747 | 0.128118 | 0.105069 | 0.106663 | -0.02116 | 0.012982 | -1.47733 | -0.08716 | -0.149 | -0.49674 | 0.006326 | 0.200361 | -0.14758 | -0.0534 |
| NCIH1944 | -0.09777 | 0.225897 | -0.04941 | 0.11104 | 0.074717 | -0.21761 | -0.96141 | 0.06607 | -0.24325 | -0.59506 | -0.07284 | 0.046813 | -0.27608 | -0.01859 |
| NCIH2023 | -0.06675 | 0.127775 | 0.045777 | 0.115739 | -0.046 | -1.14237 | -1.4711 | 0.231246 | 0.089683 | -1.05265 | -0.10375 | 0.046896 | 0.004849 | 0.012865 |
| A549 | -0.05334 | 0.08594 | -0.0233 | 0.212372 | -0.11974 | -0.08735 | -1.35522 | -0.0064 | -0.20721 | -0.63381 | 0.052072 | 0.035748 | -0.22374 | -0.20631 |
| HCC515 | -0.13823 | 0.194417 | 0.062463 | 0.250624 | -0.31333 | -0.12083 | -1.52852 | -0.2234 | -0.05042 | -0.51882 | -0.03035 | 0.002376 | -0.214 | -0.40111 |
| EKVX | -0.01918 | 0.356698 | 0.178162 | 0.18394 | -0.04151 | 0.048479 | -1.40668 | -0.0327 | 0.193338 | -0.60227 | -0.17967 | 0.179493 | 0.001336 | 0.202279 |
| NCIH1650 | -0.07592 | 0.21585 | 0.151277 | 0.223017 | 0.090676 | -0.32587 | -1.46579 | 0.153443 | -0.06838 | -0.44027 | -0.01883 | -0.02614 | -0.23699 | -0.19609 |
| HCC827 | -0.17781 | 0.255054 | -0.16158 | 0.070399 | -0.01185 | -0.06114 | -1.25725 | 0.04817 | -0.01606 | -0.7048 | -0.3567 | 0.091756 | -0.11055 | -0.0339 |
| NCIH1648 | -0.02815 | 0.156302 | 0.115024 | 0.16443 | -0.069 | 0.036043 | -1.19636 | -0.00744 | 0.054396 | -0.62166 | 0.013628 | 0.211531 | -0.14567 | -0.09045 |
| HCC827GR5 | -0.16672 | 0.00539 | 0.125107 | -0.12485 | -0.08215 | 0.005161 | -0.91006 | 0.01805 | 0.193173 | -0.60704 | 0.080976 | 0.392625 | -0.20458 | 0.025832 |
| NCIH2122 | -0.07097 | 0.285664 | 0.126475 | 0.018196 | 0.036829 | -0.09197 | -1.70843 | 0.162249 | 0.204265 | -0.52298 | -0.01429 | 0.180735 | 0.064557 | -0.18905 |
| LXF289 | -0.04921 | 0.163759 | 0.073139 | 0.030317 | -0.07229 | -0.06307 | -1.77128 | -0.11483 | -0.1488 | -0.47742 | -0.04689 | 0.132927 | -0.01869 | -0.11653 |
| NCIH1666 | 0.078597 | 0.171728 | 0.046523 | 0.077386 | -0.2346 | -0.13707 | -1.41755 | -0.06603 | -0.04603 | -0.50006 | 0.042211 | 0.189806 | -0.05598 | -0.18249 |
| HCC2935 | 0.708066 | 0.378534 | 0.097656 | 0.117255 | -0.07244 | -0.32896 | -1.29533 | 0.046028 | 0.149488 | -0.56167 | 0.064611 | 0.118281 | -0.0816 | -0.1173 |
| SW1573 | -0.25866 | 0.273592 | 0.236335 | 0.217289 | 0.106725 | 0.063567 | -1.19557 | 0.10176 | -0.33235 | -0.59104 | -0.02473 | 0.125708 | 0.023198 | -0.10624 |
| NCIH1437 | 0.005541 | 0.096813 | 0.039162 | 0.254398 | -0.07516 | -0.02914 | -1.31045 | -0.02628 | -0.25217 | -0.59343 | -0.02059 | 0.163775 | -0.19377 | -0.19815 |
| NCIH2291 | -0.24785 | 0.186823 | 0.270798 | 0.092748 | -0.00725 | -0.14 | -1.31282 | 0.040871 | 0.018368 | -0.34666 | -0.16717 | -0.02257 | 0.028735 | -0.00248 |
| A427 | -0.08211 | 0.188166 | 0.088205 | 0.126873 | -0.06995 | -0.0966 | -1.37985 | -0.16316 | 0.127603 | -0.64609 | -0.09677 | 0.231424 | -0.20302 | -0.10784 |
| PC14 | 0.14397 | 0.132534 | -0.04253 | 0.334279 | 0.005928 | -0.38104 | -0.99749 | -0.04989 | -0.03803 | -0.58766 | -0.00112 | 0.143304 | -0.01248 | -0.09108 |
| NCIH1975 | -0.03098 | 0.048827 | 0.275528 | 0.030924 | -0.057 | -0.04242 | -1.24144 | -0.09383 | 0.04456 | -0.47571 | 0.036498 | 0.041173 | -0.13203 | -0.19888 |
| ABC1 | 0.073351 | 0.071558 | 0.206875 | 0.191972 | -0.35995 | -0.6512 | -1.40339 | -0.30854 | -0.20888 | -1.23195 | -0.16547 | 0.089488 | -0.02691 | 0.068386 |
| NCIH1792 | 0.048479 | 0.22737 | -0.00359 | 0.199941 | 0.021832 | -0.06782 | -1.5953 | 0.04451 | -0.14966 | -0.35149 | 0.115758 | 0.153912 | 0.038064 | -0.12227 |
| NCIH3122 | 0.053368 | 0.167129 | -0.09092 | 0.041535 | -0.05252 | -0.02708 | -1.54162 | 0.063748 | -0.08468 | -0.38995 | -0.10829 | 0.092678 | -0.07358 | -0.15689 |
| HOP62 | -0.04127 | 0.272032 | 0.018519 | -0.08926 | -0.14355 | -0.12557 | -1.37203 | -0.20534 | -0.25277 | -0.39148 | 0.110143 | 0.067532 | -0.09805 | -0.1058 |
| HCC44 | -0.248 | 0.395255 | 0.164195 | 0.304579 | -0.19372 | -0.19354 | -1.36447 | -0.01179 | -0.29993 | -0.3532 | 0.137668 | -0.04112 | -0.14664 | 0.016071 |
| HCC461 | -0.01419 | 0.139108 | 0.159369 | 0.174387 | 0.004076 | -0.18341 | -1.47185 | 0.091308 | -0.47984 | -0.59875 | -0.13028 | 0.123705 | -0.13028 | -0.10568 |
| NCIH2030 | -0.0841 | 0.222293 | 0.032457 | 0.1393 | -0.01437 | -0.06371 | -1.63644 | 0.082195 | -0.09141 | -0.36132 | -0.03965 | 0.17219 | -0.10653 | -0.17156 |
| NCIH441 | -0.28676 | 0.305326 | 0.074726 | 0.117174 | 0.10458 | -0.4333 | -0.97901 | 0.152058 | -0.00733 | -0.78878 | -0.14332 | 0.168504 | -0.1817 | -0.2268 |
| NCIH2126 | -0.18083 | 0.159436 | 0.037745 | 0.310247 | -0.23539 | 0.007547 | -1.46208 | -0.18164 | -0.15674 | -0.65585 | -0.08408 | -0.09953 | -0.09585 | -0.06586 |
| NCIH1693 | -0.23923 | 0.056542 | 0.143663 | 0.371642 | -0.26144 | -0.11914 | -1.24701 | 0.176317 | 0.020266 | -0.49568 | -0.05644 | 0.109744 | 0.033725 | -0.11497 |
| NCIH522 | -0.29408 | 0.347602 | 0.109216 | 0.241852 | -0.22645 | -0.0133 | -1.57414 | -0.08236 | 0.004132 | -0.56689 | -0.0611 | 0.251685 | -0.12488 | -0.2567 |

CERES score approach to 0 means the gene is not an essential gene for cell survival, while score approach to -1 means the gene is an essential gene.

Abbreviations: SLC, solute carrier
